# Cis-meQTL for cocaine use-associated DNA methylation in an HIV-positive cohort show pleiotropic effects on multiple traits

**DOI:** 10.1186/s12864-023-09661-2

**Published:** 2023-09-20

**Authors:** Youshu Cheng, Amy Justice, Zuoheng Wang, Boyang Li, Dana B. Hancock, Eric O. Johnson, Ke Xu

**Affiliations:** 1 Department of Biostatistics, Yale School of Public Health, New Haven, CT 06511 USA; 2 VA Connecticut Healthcare System, West Haven, CT 06516 USA; 3 Department of Internal Medicine, Yale School of Medicine, New Haven, CT 06511 USA; 4 GenOmics, Bioinformatics, and Translational Research Center, RTI International, Research Triangle Park, NC USA; 5 Fellow Program, RTI International, Research Triangle Park, NC USA; 6 Department of Psychiatry, Yale School of Medicine, 300 George Street, New Haven, CT 06511 USA

**Keywords:** Cocaine use, Cis-methylation quantitative trait loci (cis-meQTL), Epigenome-wide association study (EWAS), Mendelian randomization, Complex trait

## Abstract

**Background:**

Cocaine use (CU) is associated with psychiatric and medical diseases. Little is known about the mechanisms of CU-related comorbidities. Findings from preclinical and clinical studies have suggested that CU is associated with aberrant DNA methylation (DNAm) that may be influenced by genetic variants [i.e., methylation quantitative trait loci (meQTLs)]. In this study, we mapped cis-meQTLs for CU-associated DNAm sites (CpGs) in an HIV-positive cohort (N_total_ = 811) and extended the meQTLs to multiple traits.

**Results:**

We conducted cis-meQTL analysis for 224 candidate CpGs selected for their association with CU in blood. We identified 7,101 significant meQTLs [false discovery rate (FDR) < 0.05], which mostly mapped to genes involved in immunological functions and were enriched in immune pathways. We followed up the meQTLs using phenome-wide association study and trait enrichment analyses, which revealed 9 significant traits. We tested for causal effects of CU on these 9 traits using Mendelian Randomization and found evidence that CU plays a causal role in increasing hypertension (*p*-value = 2.35E-08) and decreasing heel bone mineral density (*p*-value = 1.92E-19).

**Conclusions:**

These findings suggest that genetic variants for CU-associated DNAm have pleiotropic effects on other relevant traits and provide new insights into the causal relationships between cocaine use and these complex traits.

**Supplementary Information:**

The online version contains supplementary material available at 10.1186/s12864-023-09661-2.

## Background

Cocaine is one of the most commonly used illicit drugs in the United States [1], and cocaine use (CU) leads to a tremendous burden on public health and socioeconomic systems [2]. Cocaine dependence is a psychiatric disorder and also has comorbidity with numerous other psychiatric and clinical diseases [3]. CU affects the cardiovascular system and can cause palpitations, hypertension, and reduced left ventricular function, which may lead to the development of myocardial ischemia [1]. CU is also linked to pulmonary complications, including acute respiratory symptoms, airway injury, asthma, pulmonary edema, “crack lung,” and eosinophilic lung diseases [4]. Moreover, CU compromises immune function and increases the incidence of infectious diseases [5, 6]. Evidence shows that CU affects metabolism and is associated with underweight [7] and lower body mass index (BMI) [8]. The co-occurrence of CU and psychiatric disorders, such as bipolar disorder and psychosis, has been well documented [9, 10]. Although adverse consequences are widely reported, the association between genetic variation and CU’s health effects need to be better understood. While most of the genetics research on CU has focused on investigating predisposition to addictive behavior, using genetics to better understand the underlying causes of the effects of CU on complex traits is important for preventing cocaine-related comorbidities.

Previous studies have shown that cocaine administration regulates epigenetic modifications in brain and in blood [11, 12], which further regulate gene expression and cellular functions that may contribute to CU-associated complex traits. Among these epigenetic modifications, DNA methylation (DNAm) is one of the most widely studied mechanisms that capture the cumulative effects of environmental risks and heritable effects. A preclinical study by Cannella et al. showed that cocaine administration alters histone modifications and DNAm in the central nervous system in animals [13]. Shu et al*.* identified multiple CpG sites associated with persistent CU in human and showed that CU-associated DNAm is predictive of disease outcomes such as frailty in HIV infection [14]. Vaillancourt et al*.* found that CU-related hypomethylation of the *IRXA* gene cluster contributes to the development of cocaine dependence by modifying the 3D chromatin structure in the caudate nucleus [15]. Poisel et al*.* has recently reported several CU-associated differentially methylated regions (DMRs) and stressed the important role of genes *Neuropeptide FF Receptor 2 (NPFFR2)* and *Kalirin RhoGEF Kinase (KALRN)* [16]. The collective evidence highlights an important relationship between CU and DNAm.

Aberrant DNAm associated with CU can either be a consequence of cocaine exposure or be influenced by genetic variants [17]. A recent study showed that approximately 45% of DNAm sites in the human methylome are influenced by genetic variants [18]. Single nucleotide polymorphisms (SNPs) associated with DNAm are known as methylation quantitative trait loci (meQTLs) [19–22], which represent the interplay between the genome and the methylome. In recent years, genome-wide meQTL mapping has been conducted to deepen our understanding of the mechanisms of cardiovascular disease [23], smoking [24],, schizophrenia [25], and birthweight [26] However, meQTLs have not been explicitly examined for CU-associated DNAm loci and their potential role in the comorbidities linked to CU.

**Fig. 1:**
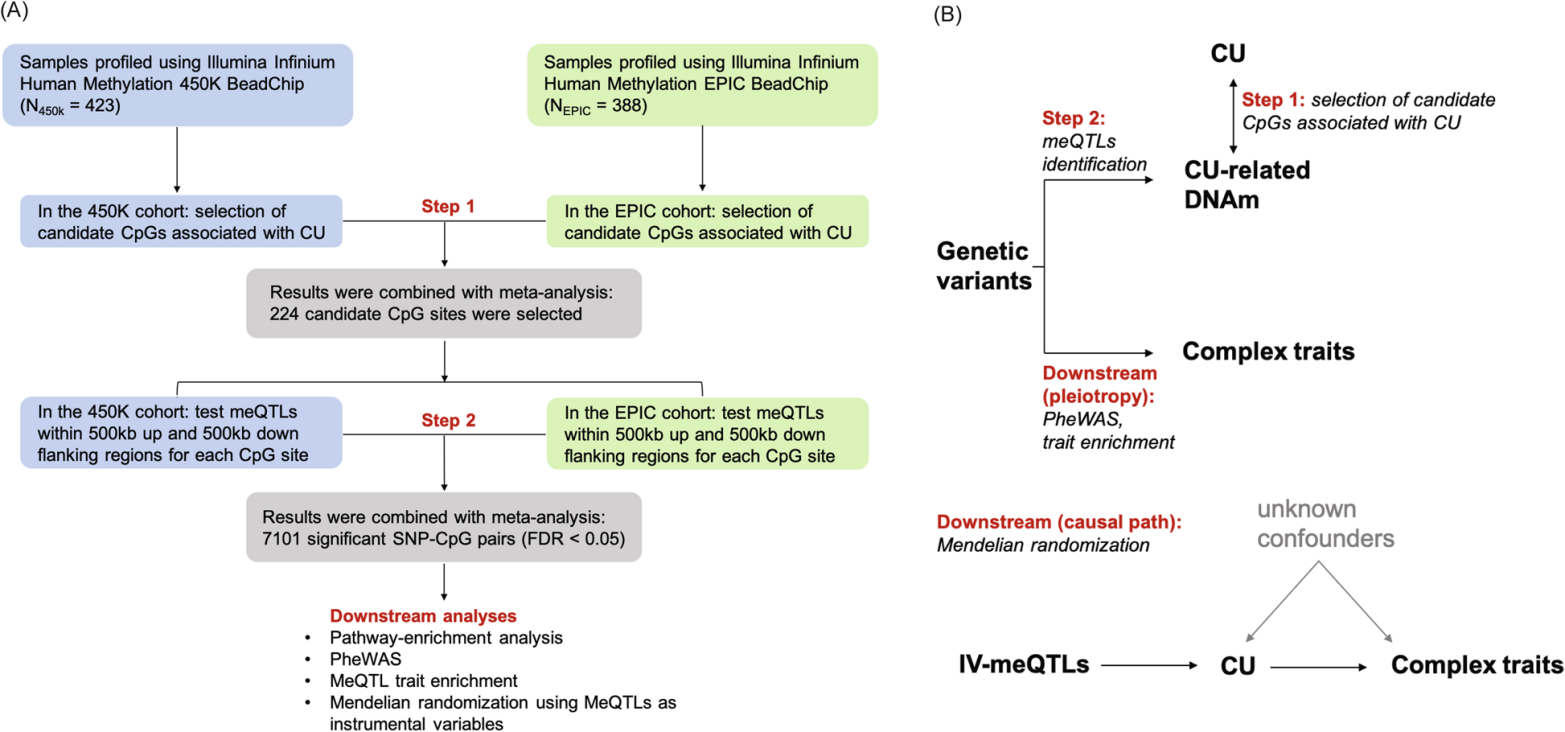
Overall study design. **A** Flowchart for the analyses performed. Selection of CU-related CpGs and meQTL identification were performed in 423 samples with DNAm profiled by 450K arrays (blue) and 388 samples with DNAm profiled by EPIC arrays (green), separately. Meta-analyses were followed to combine results (grey). Downstream analyses were conducted based on the identified meQTLs. **B** Paths involved in the analyses. After selection of candidate CpGs associated with CU (Step 1) and meQTL identification (Step 2), downstream analyses including PheWAS and meQTL trait enrichment were performed to investigate the pleiotropic effects of the meQTLs on other traits. Mendelian randomization was then performed to build causal paths. CU: cocaine use; PheWAS: phenome-wide association study; meQTL: methylation quantitative trait loci

In this study, we aimed to identify meQTLs in blood for CU-associated DNAm and then to examine the pleiotropic effects of those meQTLs on other complex traits, especially CU-related traits. Using participants from the Veterans Aging Cohort Study Biomarker Cohort (VACS-BC) (*N* = 811), we first selected candidate CpG sites for CU and then conducted a cis-meQTL analysis on the candidate CpG sites within a 1-mega-base (Mb) flanking region. We performed phenome-wide association study (PheWAS) and meQTL trait enrichment analyses to examine the pleiotropic effects of the identified meQTLs on other traits. Finally, we conducted Mendelian Randomization (MR) to further dissect the causal relationships between CU and other complex traits. The overall study design is presented in Fig. 1. In addition, we conducted a secondary analysis using frequency of CU to explore the impacts of different CU patterns on DNAm and associated cis-meQTLs.

## Results

### Participant characteristics

Study participants were from the Veteran Aging Cohort Study (VACS), a multicenter, longitudinal cohort study of the impact of substance use on HIV infection and outcomes. Instead of using socially constructed race categories, we classified a total of 2,244 participants in VACS-BC based on global ancestral information, using individuals from African (AFR), East Asian (EAS), European (EUR), and South Asian (SAS) ancestry in the 1,000 Genomes Project as our reference panel (methods)(Supplementary Fig. 1). The majority (1,622 out of 2,244, 72.3%) were estimated to be of AFR ancestry (Supplementary Fig. 2).

DNAm was profiled for a subset of those genetically defined AFR samples (N_DNAm_ = 811) by using either the Illumina Infinium Human Methylation 450K BeadChip (N_450K_ = 423) or the EPIC BeadChip (N_EPIC_ = 388) due to differing commercial availability at two different times. All 811 samples were included in the analyses. All participants were HIV-positive and were on antiretroviral therapy. Cocaine users showed higher rates for cigarette use and alcohol use than nonusers (controls) in both 450K dataset (*p* = 3.03E-12, 0.0050, respectively) and EPIC dataset (*p* = 2.47E-12, 6.91E-05, respectively) (Table 1). Combining cocaine users and controls, no significant differences in demographic or clinical variables were observed between 450K and EPIC datasets except for age (*p* = 0.01) (Table 1).

**Table 1:** Population characteristics of the HIV-positive VACS-BC participants with genotype-derived AFR ancestry

Characteristic	450K (*N* = 423)				EPIC (*N* = 388)				Pvalues for comparison between 450K and EPIC**
	**Cocaine user (*N* = 165)**	**Controls (*N* = 258)**	**Overall**	*P*value*	**Cocaine user (*N* = 128)**	**Controls (*N* = 260)**	**Overall**	*P*value*	
HIV-positive (%)	165 (100%)	258 (100%)	423 (100%)	1.0000	128 (100%)	260 (100%)	388 (100%)	1.0000	1.0000
Genotype derived ancestry-AFR (%)	165 (100%)	258 (100%)	423 (100%)	1.0000	128 (100%)	260 (100%)	388 (100%)	1.0000	1.0000
Sex-male (%)	165 (100%)	258 (100%)	423 (100%)	1.0000	128 (100%)	260 (100%)	388 (100%)	1.0000	1.0000
Age	49.2 ± 6.13	49.4 ± 8.19	49.3 ± 7.45	0.7785	48.2 ± 5.99	47.8 ± 8.88	47.9 ± 8.04	0.6229	0.0108
log10 viral load	2.70 ± 1.51	2.43 ± 1.28	2.54 ± 1.38	0.0591	2.66 ± 1.52	2.50 ± 1.22	2.56 ± 1.33	0.2975	0.8413
Antiviral medication adherence (%)	120 (73.6%)	204 (81.3%)	324 (76.6%)	0.0849	82 (66.7%)	208 (80.6%)	290 (74.7%)	0.0043	0.5941
Cigarette smoking (%)	132 (81.5%)	119 (46.7%)	251 (59.3%)	3.03E-12	99 (81.8%)	111 (42.8%)	210 (54.1%)	2.47E-12	0.1537
Alcohol use (%)	86 (52.4%)	94 (37.9%)	180 (42.6%)	0.0050	66 (53.6%)	76 (31.5%)	142(36.6%)	6.91E-05	0.0970

Mean ± SD for continuous variables and n (%) for categorical variables

^*^ *P*values were reported for the comparison between cocaine users and non-users (controls)

^**^ *P*values were reported for the comparison between overall 450K samples and overall EPIC samples

### Selection of candidate CpGs associated with CU

To avoid batch effects, the selection of candidate CpGs for CU was performed in the samples profiled by the 450K or EPIC arrays separately, and a meta-analysis of the two subsets of samples was performed to select candidate CpG sites at a meta *p* value < 0.0001.

A total of 224 candidate CpG sites for CU were selected (genomic inflation λ = 1.196) (Supplementary Fig. 3) (Supplementary Table 1). Among the 224 CpGs that were differentially methylated between CU and non-CU, 176 (78.6%) were hypomethylated, and 48 (21.4%) were hypermethylated. The 224 CpGs were mapped to their 152 nearest genes. Of them, 72 CpG sites were in promoter regions, 74 CpG sites were in gene bodies, and 12 CpG sites were in 3’UTRs. In addition, 18.8% of CpGs were in CpG islands, 10.3% were in shelves, and 19.2% were in shores. The top 2 out of the 224 CpG sites reached the epigenome-wide significance level (*p* < 1.23e-07). The top-ranked CpG, cg25508319, was mapped to the 5'UTR of *KCNJ5*, in which CU showed lower methylation levels than non-CU (*p* value = 4.41E-10). *KCNJ5* encodes inward-rectifier potassium channel proteins and could be related to exposure to cocaine via potassium channel signaling [27]. The second-ranked CpG site, cg11202380, is located on the 3’UTR of *AK2* (*p* value = 9.96E-08), which encodes adenylate kinases and is involved in regulating the adenine nucleotide composition within a cell [28].

### Identification of meQTLs for CU-associated CpG sites

We performed a cis-meQTL analysis in the 450K and EPIC samples separately, to identify genetic variants associated with the 224 candidate CU-associated CpG sites. For each CpG site, we included SNPs to those within 500 kb upstream and 500 kb downstream (overall 1 Mb flanking regions). After a meta-analysis to combine results and FDR correction, clumping was performed to group correlated meQTLs (LD > 0.1) into one clumped region for each CpG site, and the most statistically significant meQTL for each region was selected as the index meQTL.

**Fig. 2:**
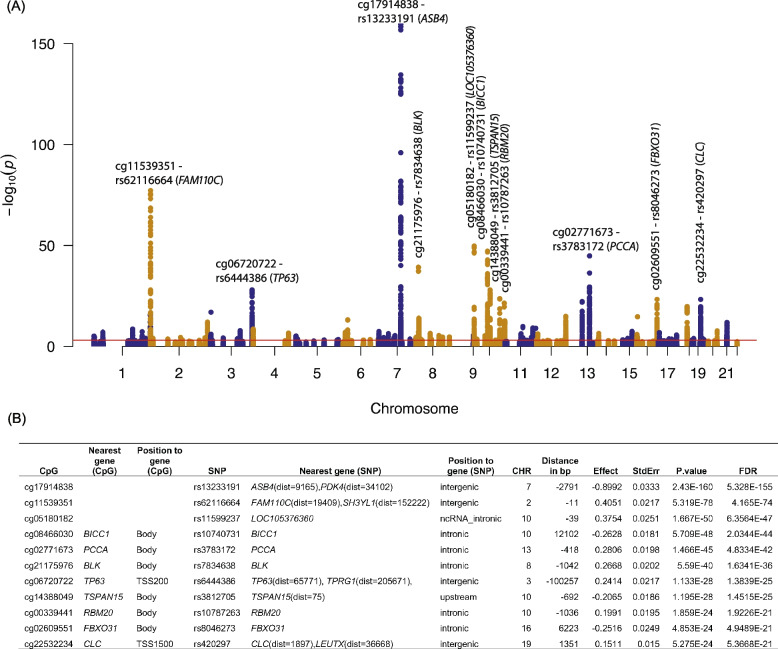
Cis-meQTLs identified for cocaine use-associated CpG sites. **A** Manhattan plots of meQTL results following meta-analysis. The red line indicated the FDR-corrected 0.05 level (*p*-value = 8.9e-4). The top SNP (index meQTL) in the most significant meQTL clumps was marked with the corresponding CpG site and gene. **B** The top 11 index meQTLs marked in (**A**). meQTL: methylation quantitative trait loci

A total of 7,101 SNP-CpG pairs surpassed the significance threshold (FDR < 0.05). After grouping the highly correlated meQTLs based on LD, we identified 448 index meQTLs for 124 CpG sites associated with CU (Fig. 2) (Supplementary Table 2), suggesting that approximately 55% of CU-associated CpGs were influenced by genetic variants. Index meQTLs were found on each of the 22 autosomal chromosomes and were mapped to their 250 nearest genes. Most index meQTLs were in intergenic (199 out of 448, 44.42%) or intronic (168 out of 448, 37.50%) regions of their corresponding genes. Among the 448 index meQTLs, 222 (49.6%) were associated with increased methylation at the corresponding CpG site, while 226 (50.4%) were associated with decreased methylation at the CpG. Supplementary Fig. 4 displays two SNP-CpG pairs as examples. For the rs13233191-cg17914838 pair near *ASB4* on chromosome 7, the effect allele (G) of the SNP decreased the methylation level of cg17914838 (FDR = 5.33E-155) (Supplementary Fig. 4A). For the rs7834638-cg21175976 pair on *BLK*, the effect allele (C) of the SNP increased the methylation level at cg21175976 on chromosome 8 (FDR = 1.6341E-36) (Supplementary Fig. 4B).

The top index meQTL was rs13233191 (FDR = 5.33E-155), located near *ASB4*, a gene involved in the innate immune system and class I MHC-mediated antigen processing and presentation. There were 35 additional meQTLs in the MHC region, including *HLA-A, HLA-B, HLA-G, HLA-DQB2, and HLA-DMB*. The top CpG for CU (cg25508319 in *KCNJ5*) was significantly associated with the rs582823 variant (FDR = 0.036), while the second-ranked significant CpG site (cg11202380 in *AK2*) was not associated with any variant. The results suggest that genetic variants impact more than half of CU-associated methylation sites, while a proportion of CU-associated CpG sites likely could result from cocaine exposure.

### Pathway-enrichment analyses based on identified meQTLs

Using QIAGEN Ingenuity Pathway Analysis (IPA) to perform pathway enrichment analyses on genes mapped by the identified meQTLs [29], we identified 9 ingenuity canonical pathways (FDR < 0.05) (Supplementary Table 3). The top pathways were (1) antigen presentation (FDR = 2.03E-05), which included *HLA-A, HLA-B, HLA-C, HLA-DMB, HLA-DOB, HLA-DQB2, HLA-G,* and *PSMB5*; (2) PD-1/PD-L1 cancer immunotherapy (FDR = 4.13E-04); (3) the xenobiotic metabolism AHR signaling pathway, which included genes for smoking (*AHRR*), alcohol metabolism (*ALDH16A1*) and histone modification (*HDAC4*); and (4) crosstalk between dendritic cells and natural killer cells (FDR = 3.75E-03), which included genes for immunity (HLA family) and inflammation (*TNF*). These significant pathways are involved in immunity-related functions and activities.

### PheWAS analysis of meQTLs for CU-associated CpG sites

**Fig. 3:**
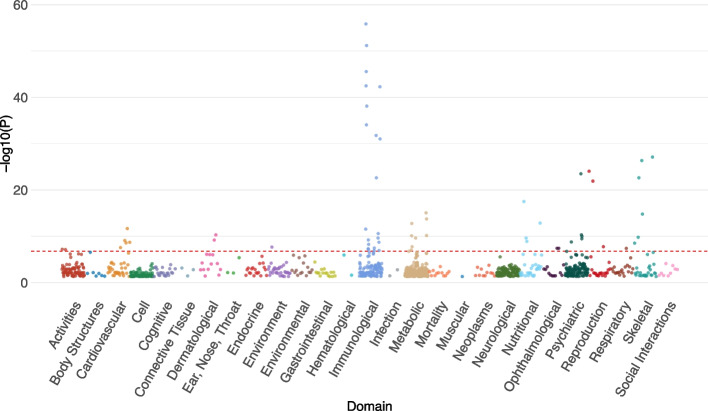
Manhattan plot of the traits associated with the top index meQTLs in PheWAS analysis. The associations and classification of traits (domains) were obtained from 4,756 GWAS studies available on GWAS Atlas. The red dash line indicated the Bonferroni-corrected 0.05 level (*p*-value = 1.70E-07). PheWAS: phenome-wide association study; meQTL: methylation quantitative trait loci

To investigate pleiotropic effects, we performed a PheWAS on the top 62 meQTLs (meta *p* value < 5E-8) for CU-associated CpGs (Methods). Using the GWAS Atlas database consisting of SNP-trait association results from 4,756 genome-wide association studies (GWASs) [30], PheWAS identified 36 significant traits (p_Bonferroni_ < 0.05) (Supplementary Table 4). The 36 significant traits were in the following functional domains (Fig. 3): immunological (10 traits), metabolic (6 traits), nutritional (4 traits), skeletal (3 traits), cardiovascular (3 traits), psychiatric (2 traits), reproduction (2 traits), activities (2 traits), ophthalmological (1 trait), dermatological (1 trait), respiratory (1 trait), and environment (1 trait). In the immunological domain, meQTLs for CU-associated CpGs were implicated for eosinophil count (rs1034867 near *CLC*, *p* = 2.92E-12) and hemoglobin concentration (rs10740731 in *BICC1*, *p* = 5.64E-09). In the category of cardiovascular disease, the meQTL rs10740731 in *BICC1* was previously implicated in hypertension (*p* = 2.8E-08) [31]. Two meQTLs, rs4785958 (near *TFAP4*, *p* = 2.1E-12) and rs56218021 (in *DLC1*, *p* = 3.2E-08), were associated with resting heart rate [31]. In the metabolic domain, several meQTLs were previously associated with BMI (rs7834638 in *BLK*, *p* = 1.7E-13; rs1078763 near *FAM110C*, *p* = 3.1E-09; rs9952447 near *SKOR2*, *p* = 9.22E-09) [32–34]. Of note, one of the top-ranked significant meQTLs, rs7834638 in *BLK*, was previously associated with multiple cocaine-related phenotypes, such as BMI (*p* = 1.7E-13) [32, 33], neuroticism (*p* = 4.62E-11) [35, 36] and bone mineral density (*p* = 4.5E-27) [37–39]. Our PheWAS results identified variants with pleiotropic effects on both CU-associated DNAm and other phenotypes relevant to CU.

### MeQTL trait enrichment

**Fig. 4:**
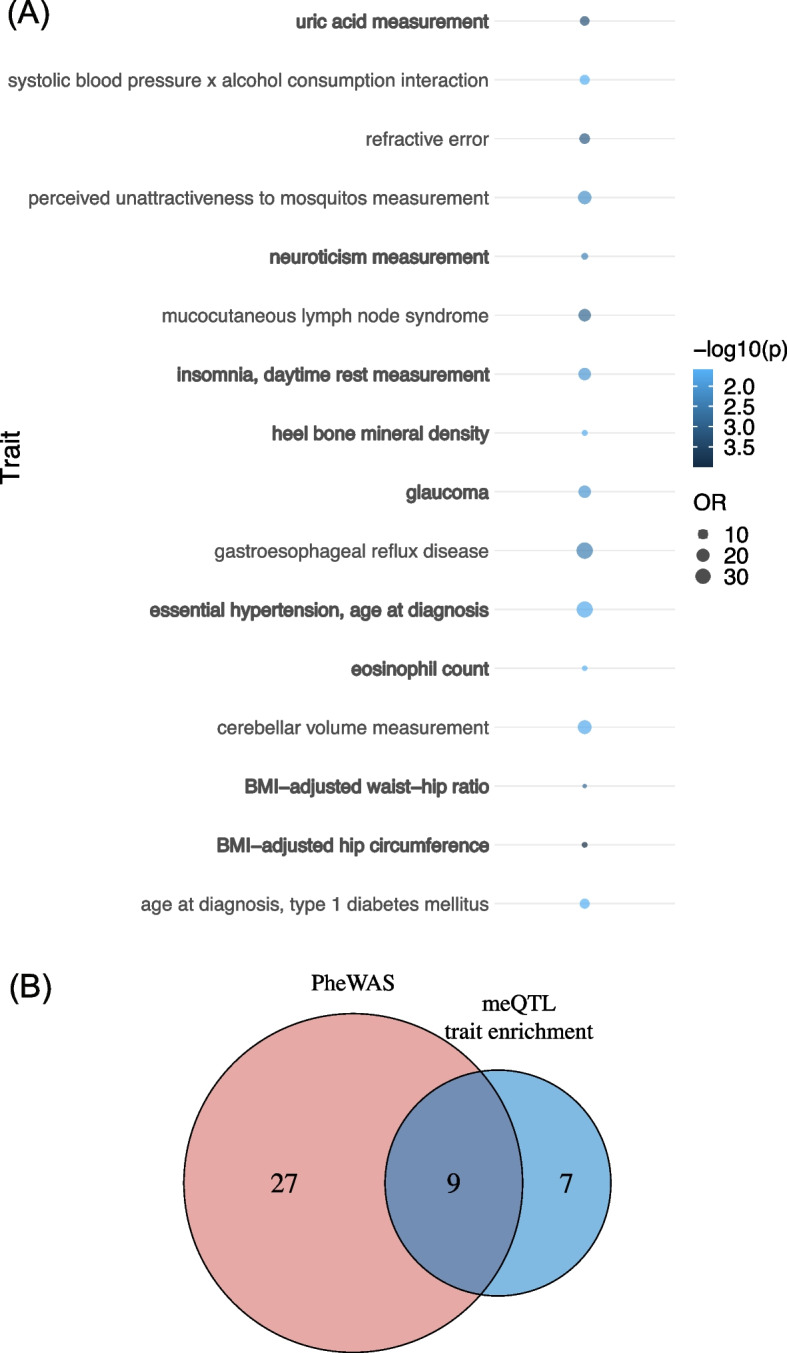
Results of meQTL trait enrichment analysis. **A** Dot plot for the 16 complex human traits which the meQTLs were enriched in (*p*-value < 0.05). **B** Venn plot to compare the 36 phenotypes identified by PheWAS and 16 human traits identified by meQTL trait enrichment. The 9 overlapped traits were marked in bold in (**A**). PheWAS: phenome-wide association study; meQTL: methylation quantitative trait loci

PheWAS analysis identified traits that were associated with the index meQTL by examining one variant at a time. To systematically reveal the pleiotropic effects of meQTLs, we performed an enrichment analysis to identify traits whose risk variants were enriched with meQTLs for CU-associated CpGs (Methods). Using data from the GWAS Catalog (https://www.ebi.ac.uk/gwas/) and Fisher's exact test to declare statistical significance [40], we observed that the meQTLs were enriched for 16 traits (*p* value < 0.05) (Fig. 4A). The top two traits were BMI-adjusted hip circumference (OR = 2.73, *p* value = 1.01E-4) and uric acid measurement (OR = 8.47, *p* value = 5.28E-4) (Supplementary Table 5).

Of the 16 significant meQTL-enriched traits, 9 overlapped with traits implicated from the PheWAS results: hip circumference (OR = 2.73, *p*-value = 1.01E-4), uric acid measurement (OR = 8.47, *p*-value = 5.28E-4), waist-hip ratio (OR = 2.27, *p*-value = 1.46E-3), neuroticism (OR = 3.64, *p*-value = 4.28E-3), glaucoma (OR = 17.52, *p*-value = 0.0091), insomnia (OR = 17.52, *p*-value = 0.0091), hypertension (OR = 34.09, *p*-value = 0.0187), heel bone mineral density (OR = 2.87, *p*-value = 0.0221) and eosinophil count (OR = 2.54, *p*-value = 0.0247) (Fig. 4B). Of note, there has been accumulating evidence for the association between CU and sleep abnormalities [41, 42], hypertension [1, 43], and glaucoma [44]. Thus, the trait enrichment results, combined with the PheWAS results, further suggest the pleiotropic effects of genetic variants on CU-associated DNAm and other complex traits relevant to CU.

### Mendelian randomization using MeQTLs as instrumental variables

To take a step further from pleiotropy to inferring causal relationships between CU and other traits, we performed Mendelian randomization (MR) for the 9 traits identified by both PheWAS and meQTL trait enrichment. We used meQTLs that were significantly associated with CU (*p* value < 0.05) as instrumental variables (referred to as IV-meQTLs) (Methods), CU as the exposure, and other traits as outcomes. Summary statistics for the 9 traits from 13 publicly available GWASs were used for the MR analysis with inverse-variance weighted (IVW), weighted median (WM), and MR-PRESSO. Bonferroni correction was applied to correct for different MR methods. We present the causal paths for statistically significant CU-trait pairs identified by at least one of the three MR methods.

We identified significant causal paths for 3 of the 9 tested traits: hypertension, heel bone mineral density and neuroticism (Table 2). For example, IV-MeQTL-driven CU could cause hypertension (*p* value = 2.35E-08 in MR-PRESSO). All the three MR methods also indicated that CU that driven by IV-MeQTLs decreased heel bone mineral density (*p* value = 4.78E-12 in IVW, *p* value = 2.79E-11 in WM, and *p* value = 6.9E-19 in MR-PRESSO).

**Table 2:**
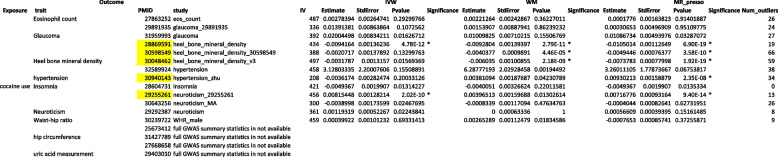
Mendelian randomization (MR) results

Mendelian randomization (MR) analysis was performed with CU as exposure, 9 other traits as outcome, and meQTLs that were significantly associated with CU as instrumental variables (IV-meQTLs). For the 9 traits included, a total of 13 studies with available GWAS summary statistics were tested and each of them by 3 methods, resulting in a significance cutoff 0.05/13/3 = 1.28E-3

We marked significant causal paths identified by one of the three MR methods in yellow

### Assessment of frequency of CU among cocaine users

Intensity of drug use could have impacts on the DNAm alterations [45]. To better assess the effects of different CU patterns among our cocaine user group, we performed a secondary analysis by comparing DNAm levels between high-frequency users (self-reported CU at least once a month) and low-frequency users (self-reported CU less than once a month) (methods). We found 141 candidate CpGs (Supplementary Table 6) and 3,356 significant meQTLs (FDR < 0.05), which were grouped into 146 index meQTLs based on LD (Supplementary Table 7). Subsequently, pathway analysis for the identified meQTLs did not reveal significant pathways at FDR < 0.05 (Supplementary Table 8). The top pathway, PD-1/PD-L1 cancer immunotherapy (FDR = 0.12), was identified in our main analysis for the CU phenotype (FDR = 4.13E-04) (Supplementary Table 3). The PheWAS analysis identified 4 significant traits: estimated glomerular filtration rate, Type 2 Diabetes, FVC, and age at menarche (Supplementary Table 9), and the meQTL trait enrichment analysis identified 2 traits: susceptibility to plantar warts measurement and balding measurement (Supplementary Table 10). Of note, in our main analysis there were 9 traits identified by both PheWAS and meQTL trait enrichment and then MR models were built for them, but here the PheWAS and trait enrichment for CU-frequency did not have overlapped results. Overall, cis-meQTL identification and downstream analyses resulted in fewer signals and did not reveal additional information than our main analysis contrasting CU with non-users.

## Discussion

This study identified 224 CU-associated CpG sites and their corresponding cis-meQTLs in an African ancestry population. Our results showed that approximately 55% of CU-associated CpG sites in blood were influenced by nearby genetic variants. The set of meQTLs identified for CU-associated CpGs had pleiotropic effects on complex traits previously linked to CU, such as immunological, cardiovascular, metabolic, and psychiatric traits. Using meQTLs as instrumental variables, we further found causal relationships between CU and 3 traits. These findings provide new insights into the underlying mechanisms of CU and its relevant conditions.

A recent study on genome-wide cis- and trans-meQTLs reported that up to 45% of CpG sites in blood were influenced by genetic variants [46]. Similar to this report, we identified significant cis-meQTLs for 55% of candidate CpG sites for CU (124 out of 224 CpG sites). The top-ranked CpG, cg25508319 on *KCNJ5,* was significantly associated with the rs582823 variant (FDR = 0.036)*.* Genetic variants in potassium channel signaling, such as *KCNJ9,* were previously linked to cocaine dependence [47]. Also, Bao et al*.* has reported that *KCNJ* genes are involved in multiple pathways that contribute to the pathophysiology of major depressive disorder [48], which is a psychiatric disorder frequently comorbid with cocaine use disorders [49]. The second-ranked CpG site, cg11202380 on *AK2,* was not influenced by any variant in our cis-meQTL mapping. A previous study showed that cocaine administration in rats upregulates *AK2* expression in Drd2 DA receptor–positive medium spiny neurons [50]. Together, the evidence indicates that half of the CU-associated DNAm sites could be affected by genetic variants (e.g., cg25508319 on *KCNJ5*). In contrast, the remaining CU-associated DNAm sites may reflect a biological consequence of cocaine exposure (e.g., cg11202380 on *AK2).* Comparing our findings with one previous study on the association between persistent CU and DNAm in human [14], we found that four of our CU-associated CpGs were also reported by them: cg23058613 on *GANAB*, cg12444450, cg09490603 on *CYP11A1*, and cg15486123 on *IVL*. Of note, Marceau et al*.* has shown that the gene *CYP11A1* was significantly associated with glucocorticoid action, which might further impair neurogenesis in the hippocampus and confer vulnerability to substance use [51]. Other CU-associated CpGs worth noting includes cg09659661 on *IRX6*, which has been supported by other studies showing that the hypomethylation of *IRX* genes contributes to the development of cocaine dependence [15]. Additionally, cg09356524 on *ZMYND8* has been identified as one of the top CpGs associated with drug injection intensity (heroin or cocaine) [45], and we also identified a nearby site cg03082779 on *ZMYND8* as a CU-associated CpG.

MeQTL and pathway analyses highlighted loci related to immunological function. It has been well established that cocaine exposure compromises immune function and increases the risk of infectious disease. Cocaine exposure has significant effects on T, B, and natural killer cells through the interaction of dopamine receptors on immune cells that are involved in the regulation of cellular processes such as apoptosis, proliferation, and differentiation [52]. An increase in the proinflammatory cytokines *IL6* and *TNF* and a decrease in anti-inflammatory cytokines have been reported in cocaine users [53]. Consistent with the immunosuppressive effects of cocaine administration, our meQTLs identified in or near immune genes may add more molecular targets to the list of developing or repurposing therapies for treating cocaine use disorder.

PheWAS suggested pleiotropic effects of meQTLs on several complex traits. For example, rs7834638 was found to be a risk variant influencing neuroticism [23], and a high neuroticism score was found to be independently associated with the psychotic symptoms induced by cocaine [25]. Similarly, rs10740731 in *BICC1*, one of the top index meQTLs, was found to be associated with hypertension [30, 31] and open-angle glaucoma [54]. We found that 9 of 36 PheWAS-significant traits were consistent with meQTL trait enrichment analysis, including hypertension, insomnia, heel bone mineral density, glaucoma, neuroticism, eosinophil count, hip circumference, uric acid measurement, and waist-hip ratio. The results suggest that meQTLs for CU-associated CpGs have pleiotropic effects on these 9 phenotypes. We further investigated the causal relationships of CU on 9 traits using the meQTLs as instrumental variables. For example, IV-MeQTLs showed that CU could lead to hypertension, which is consistent with the clinical observation that cocaine is a powerful vasoconstrictor and that its vasotoxicity induces a variety of cardiovascular effects [55]. Together, these findings suggested that genetic variants for CU-associated DNAm not only have pleiotropic effects on other relevant traits but also could serve as instrumental variables to build the causal path from CU to complex traits, for example, cardiovascular disease as evidenced in this study.

Although our main interest was on the cocaine exposure and we aimed to identify meQTLs for CU-associated DNAm, we also conducted a secondary analysis for the frequency of CU phenotype (high-frequency users versus low-frequency users). This was motivated by previous studies showing that frequency of drug use was associated with DNA methylation alterations [45]. Compared to the main results contrasting CU versus non-CU, we found fewer candidate CpGs and meQTLs in the secondary analysis, and the downstream analyses also did not add more information. One potential reason is the reduced sample size from 811 (N_450K_ = 423, N_EPIC_ = 388) to 293 (N_450K_ = 165, N_EPIC_ = 128) by excluding non-users. Another explanation would be the potential errors associated with self-reported data on usage frequency among cocaine users. In the future, applying biomarker to obtain more accurate measurements of usage frequency will help to better explore the CU frequency phenotype and increase the power for candidate CpGs and cis-meQTL identifications.

We acknowledge several limitations of our study. First, in the step of selecting candidate CpGs, our sample size limited the identification of a large number of epigenome-wide significant CpGs associated with CU. Instead, a liberal *p*-value cutoff was used to maintain a reasonable pool of candidate CpGs for further analysis, preventing an excessively small number of CpGs in the subsequent meQTL mapping [14]. As recommended [56, 57], a dataset with larger sample size (over 1,000) and more balanced design (50% cases, 50% controls) can benefit future studies with more CU-associated CpGs reaching epigenome-wide significance. Second, although we combined 450K and EPIC cohorts using meta-analysis, an independent cohort will be helpful to further replicate and validate our findings. Third, our significant results regarding pleiotropy were based on comparisons between meQTLs identified in an African ancestry population and other published GWASs that were mostly derived from European ancestry populations (due to the fact that the vast majority of available GWAS were performed in European ancestry populations [58, 59]). Although this might indicate the transferability and stability of our identified meQTLs, it is still worth verifying whether our findings could be replicated in European ancestry populations. It is also noted that the identified significant MR results served as suggestive evidence, and the causal relationships should be interpreted with caution. Although we tried to detect outliers of instrumental variables that may exert horizontal pleiotropic effects by MR-PRESSO and provide corrected estimates via outlier removal, further investigations using longitudinal designs in larger samples would be helpful to validate the causal relationships that we identified here [17, 39, 40]. Other than MR paths, it will also be interesting to use mediation models to investigate the mediation roles of DNAm in the future studies. Furthermore, we focused on the effects of cis-meQTLs on CU-related epigenetic regulation. The effects of trans-meQTLs should also be examined in future studies. Last, our analysis focusing on peripheral blood DNAm was not designed to inform brain mechanisms of cocaine addiction. However, given that Qi et al*.* has reported that the genetic effects on DNAm between the blood and the brain are correlated [60], extending our findings from the blood to the brain could merit future study once more data are available on DNAm profiles in brain tissues.

## Conclusions

In summary, the study identified meQTLs for CU-associated DNAm in an African ancestry population with HIV positive status and suggested that leveraging epigenome-wide methylation data and genetic variants can help to establish potential causal paths from CU to other relevant complex traits. Further validation with larger and more diverse sample sizes will shed more light on the molecular mechanisms of genetic and epigenetic risks for CU.

## Methods

### Study samples and cocaine-related traits

The Veterans Aging Cohort Study (VACS) was a prospective, observational cohort study to investigate substance use and HIV-related outcomes with electronic medical records and biospecimen data [61]. After the baseline survey at enrollment, 5 follow-up visits occurred at approximately 1-year intervals. Blood samples were collected in the middle of the follow-up period for a subset of participants (VACS-BC) [14]. Ancestry estimation by genotype was carried out for a total of 2,244 participants in the VACS-BC. A total of 811 DNA samples from individuals of African ancestry with both genotyping and DNAm data available were used for this study. The CU group was defined based on self-reported CU within the 12 months preceding blood sample collection, and the nonuser group (control) was defined based on self-report of no cocaine exposure. The usage of other substances (tobacco and alcohol) was also assessed in the CU group and control group. Among CU users (*N* = 165 in the 450K dataset, and 128 in the EPIC dataset), we further defined individuals who self-reported using cocaine at least once a month as high-frequency users (*N* = 113 in the 450K dataset, and 88 in the EPIC dataset), while those who reported less frequent use were categorized as low-frequency users (*N* = 52 in the 450K dataset, and 40 in the EPIC dataset).

### Genotyping and quality control

The VACS samples were genotyped using the Illumina HumanOmniExpress BeadChip. Imputation was performed using IMPUTE2 (ver 2.3.2) with the 1000 Genomes Phase 3 as the reference panel [62], yielding 18.5 million variants. Quality control (QC) was conducted by removing SNPs with a minor allele frequency < 0.05, a missing rate > 0.05, an imputation quality r2 < 0.8, and those that deviated significantly from Hardy–Weinberg equilibrium (*p* < 1e-6). A total of 5.1 million variants passed QC and were used for global ancestry estimation and meQTL identifications.

### DNA methylation profiling

DNAm was profiled at different time frames using either the Illumina Infinium Human Methylation 450K BeadChip (N_450k_ = 423) or the Infinium Human Methylation EPIC BeadChip (N_EPIC_ = 388) [14]. Two subsets of samples were processed in different periods but were processed using the same procedure at the Yale Center for Genomic Analysis [63]. We followed the procedure described in Lehne et al*.* for methylation normalization and batch effect adjustments [64]. We also removed the polymorphic CpG sites (the ones that overlay with SNPs) and CpG sites containing SNPs within 10 base pair (bp). Additionally, following the previous report [64], we removed CpG sites with detection *p*-value > 1e-12, a more stringent threshold than recommended by Illumina (*p* > 0.01). The use of a stringent detection *p*-value could more effectively filter out poor quality CpG sites and enhance the quality of DNAm array data [65]. A total of 407,793 CpG sites covered by both 450K and EPIC arrays passed QC steps and were used in the analyses. We applied the method described by Houseman et al. to estimate the cell type proportions for CD4 + T cells, CD8 + T cells, natural killer cells, B cells, monocytes, and granulocytes [66, 67].

### Global ancestry estimation

To estimate the genotype-based global ancestral information for VACS samples, 2,504 residents from the 1000 Genomes Project were used as the reference genotype panel to infer membership in the superpopulations (AFR: African, EAS: East Asian, EUR: European, and SAS: South Asian ancestry) [68]. The overlapping SNPs between the VACS and the 1000 Genomes Project were kept in the analysis. Pruning was performed by PLINK with linkage disequilibrium (LD) measured r^2^ set to 0.02, yielding 57,303 SNPs. Principal component analysis (PCA) and ADMIXTURE with the number of ancestral groups set to 4 were performed on this set of SNPs to visualize the VACS population structure and to estimate the individual-level global proportions for the 4 ancestral groups (AFR%, EUR%, EAS%, SAS%). We aimed to cluster the admixed individuals into another special group with admixed ancestry information. By comparing the ADMIXTURE results and further calculating the individual-level minimal Euclidean distance to the 4 reference groups’ centroids of genotypes PC1 to PC3, we identified 105 VACS samples with admixed genetic ancestral compositions (Supplementary Fig. 1B). To further map VACS samples to the reference ancestral groups, we applied the k-nearest neighbor (KNN) algorithm with k set to 20. The following variables were included: genotypes PC1 to PC10 and proportions for the 4 ancestral groups from ADMIXTURE. The training set was the 4 reference groups plus 1 representative admixed group consisting of 20 samples randomly selected from the 105 admixed VACS samples. The testing set was the remaining VACS samples (2224). The estimated genotype-based global ancestry was used in all the downstream analyses.

### Selection of candidate CpGs associated with CU

Cocaine-associated DNAm was identified in the 450K and EPIC subset cohorts (N_450k_ = 423, N_EPIC_ = 388) separately. In each of the two cohorts, we first applied a linear regression on the methylation M-value against the following covariates: age, tobacco use, alcohol consumption, log_10_ of viral load, antiviral medication adherence, white blood count, estimated cell-type proportions, and the top 20 PCs on DNA methylation levels measured at control probes. We then applied a second linear regression on the methylation M-value against the outcome variable of interest (CU), including all the above covariates and the top 5 PCs on residuals from the initial model to capture the remaining batch effects [69]. We used M to represent the candidate CpG methylation M value, Y to represent CU (cocaine user vs. nonusers), and C_i_ to represent k covariates (i = 1, 2…k). The model used to identify candidate CU-associated CpGs, was as follows:

$$\mathrm{M }={\alpha }_{0}+{\alpha }_{1}{Y}+{\sum }_{i=1}^{k}{\alpha }_{i+1}{C}_{i}$$

To combine results from the 450K and EPIC cohorts, we performed fixed-effects, inverse-variance weighted meta-analysis with METAL [70]. Candidate CpG sites were selected at a meta *p* value < 0.0001. We set a liberal selection threshold to ensure that there would be a sufficient number of candidate CpGs for meQTL identification [14].

We also selected candidate CpGs associated with CU frequency (high-frequency users versus low-frequency users). The procedure was the same and the results were summarized in Supplementary Table 6.

### Identification of meQTLs for CU-associated CpG sites

To identify genetic variants that influence DNAm levels at CU-associated CpG sites, we mapped meQTLs in VACS samples profiled by the 450K and EPIC arrays separately. In each cohort, meQTLs were tested by constructing linear regression models of methylation M values at each candidate CpG site on SNP genotypes from 500 kb upstream to 500 kb downstream, and analysis was performed using FastQTL [71]. We used M to represent the candidate CpG methylation M value, X to represent genotype, and C_i_ to represent k covariates (age, tobacco use, alcohol consumption, log_10_ of viral load, antiviral medication adherence, white blood count, estimated cell-type proportions, and the top 20 PCs on DNA methylation levels of control probes). The meQTL model was as follows:

$$\mathrm{M }={\beta }_{0}+{\beta }_{1}X+{\sum }_{i=1}^{k}{\beta }_{i+1}{C}_{i}$$

A fixed-effects, inverse-variance weighted meta-analysis was conducted to combine results from the 450K and EPIC cohorts. After multiple test corrections with false discovery rate (FDR) [72], SNP-CpG pairs were selected at an FDR < 0.05. Considering the LD between variants, we performed clumping on the meQTLs identified for each CpG site. Highly correlated genetic variants were clustered into one clump with an LD r2 > 0.1, and the lead SNP was identified as the one with the smallest *p* values in a clump. The lead SNP in one clump was identified as the index meQTL for the corresponding CpG site. The same procedure was followed to identify genetic variants that influence DNAm levels associated with CU frequency.

### Pathway-enrichment analyses based on identified meQTLs

We used ANNOVAR to map variants to their nearest gene, and for variants in intergenic regions, the closest gene was kept [73]. Using the 7,101 identified meQTLs (FDR < 0.05), we obtained a total of 410 genes. Pathway enrichment analyses were conducted with QIAGEN Ingenuity Pathway Analysis (IPA) (QIAGEN Inc., https://digitalinsights.qiagen.com/IPA) [29]. We identified significant pathways at FDR < 0.05. We also performed the same procedure on the 3,356 identified meQTLs for CU frequency associated CpG sites.

### PheWAS analysis

Consistent with other studies that kept only genome-wide significant, independent SNPs for PheWAS analyses [74–76], we analyzed the phenotype associations of the top 62 index meQTLs (meta *p* value < 5E-8) using the available PheWAS database in the GWAS Atlas, which consists of 4,756 GWAS studies [30]. For each variant, the list of associated phenotypes (*p* < 0.05) was collected. With the candidate list of PheWAS hits compiled from all 62 variants, the significant phenotypes associated with top index meQTLs were selected after Bonferroni correction (*p* < 0.05/62/4756 = 1.70E-07) [76, 77]. The same procedure was also applied on the top index meQTLs (meta *p* value < 5E-8) for CU frequency associated CpG sites.

### MeQTL trait enrichment

To systematically examine whether the identified meQTLs were enriched for other diseases/traits, especially those relevant to CU, we performed meQTL trait enrichment analysis using Fisher's exact test [78, 79]. A 2 × 2 contingency table was built as follows:

**Table:** 

	**meQTLs**	**Genome (non-meQTLs)**	Total
**Associated with trait D**	MR	R - MR	R
**Not associated with trait D**	M – MR	T - R - (M - MR)	T - R
**Total**	M	T - M	T

The total sum of the 2 × 2 contingency table (T) was the number of overall variants involved in the meQTL identification step. We used associations downloaded from the GWAS Catalog (https://www.ebi.ac.uk/gwas/) to decide whether a variant belonged to the list of risk variants (R) that were associated with a certain trait or not (T—R) [40]. Variants with a meta-FDR < 0.05 in the meQTL identification step were defined as meQTLs (M), and the count of overlapped variants between the list of meQTLs and the list of risk variants was reflected in the upper-left category of the table (MR). The remaining 3 categories of the table were then calculated based on MR and the row/column sums. Based on the 2 × 2 contingency table, we tested whether the probability for the meQTLs to be associated with trait D was more often than random chance compared to the genome background. We used a Fisher's exact test *p* value < 0.05 to conclude whether the meQTLs were associated (enriched) with the risk variants of trait D more often than random chance [80, 81].

### Mendelian randomization using meQTLs as instrumental variables

We performed Mendelian randomization (MR) with CU as the exposure and the other traits identified by both PheWAS and meQTL trait enrichment as the outcomes, which consisted of 9 traits with 13 publicly available GWASs. Summary statistics were downloaded from the NHGRI-EBI GWAS Catalog [82] for the 13 studies [31, 33, 35–39, 54, 83–87]. Among the 7,101 meQTLs (meta-FDR < 0.05), PLINK (v1.9) was employed to perform linear regression and test the association between them and CU [88], resulting in 503 meQTLs that were potential risk loci for CU (*p* value < 0.05). The instrumental variables were selected as those 503 variants (referred to as IV-meQTLs) to satisfy the MR assumptions. Three MR methods were used: IVW and WM implemented in MendelianRandomization (v0.4.2) and MR-PRESSO implemented in MR-PRESSO (v1.0) [89–91]. MR-PRESSO was also employed to identify the outliers of instrumental variables with horizontal pleiotropic effects, and we reported causal estimates from MR-PRESSO after removing the outliers. Bonferroni correction was applied on the 13 pairs, each of which was tested by 3 methods. Thus, the final significance cutoff was 0.05/13/3 = 1.28E-3.

### Supplementary Information

**Additional file 1:** **Supplementary Figure 1.** Global ancestry estimates by ADMIXTURE. 2,504 residents with African (AFR), East Asian (EAS), European (EUR), and South Asian (SAS) ancestry from the 1000 Genomes Project were used as the reference genotype panel to infer the super populations membership. Results were plotted for (A) samples in the Veterans Aging Cohort Study (VACS) cohort (*n* = 2244) with respect to reference samples, (B) a subset of the VACS cohort with admixed ancestral information (*n* = 105) with respect to reference samples. The reference and VACS samples were separated by the black line. **Supplementary Figure 2.** Inferred global ancestry of the VACS samples. Scatter plot of the genotype principal component analysis (PCA) results (PC1 and PC2) for the VACS cohort and 1000 Genome Project were plotted. The color indicated the super population of 1000 Genome reference samples (dots), and the inferred global ancestry of the VACS samples (triangles). **Supplementary Figure 3.** Selection of candidate CpGs associated with cocaine use in the Veterans Aging Cohort Study (VACS) samples. (A) Manhattan plot and (B) QQ plot (genomic inflation λ = 1.196) after meta-analysis to combine results from the 450K and EPIC cohorts. A total of 224 candidate CpG sites were identified. The red line indicates the *p*-value threshold used to identify candidate CU-associated CpG sites (*p*-value < 0.0001). **Supplementary Figure 4.** Two representative patterns of genetic effects by cocaine use for the meQTLs identified. (A-B): the distribution of methylation by the genotype among cocaine non-users and users. The patterns in the 450K cohort and EPIC cohort were plotted separately. (A) The genetic effect of rs13233191 on the methylation of cg17914838. (B) The genetic effect of rs7834638 on the methylation of cg21175976. CU: cocaine use; meQTL: methylation quantitative trait loci.

**Additional file 2:** **Supplementary Table 1.** Candidate CpG sites for cocaine use after meta-analysis to combine results for the 450K and EPIC cohorts (Ntotal = 811). **Supplementary Table 2.** Selected SNP-CpG pairs (FDR < 0.05) after meta-analysis to combine results for the 450K and EPIC cohorts (Ntotal = 811) and clumping. **Supplementary Table 3.** Enriched Ingenuity Canonical Pathways identified using genes mapped by the meQTLs for cocaine-related DNA methylations. **Supplementary Table 4.** Significant traits associated with the top index meQTLs. A total of 60 studies reached the significance level after bonferroni correction (p < 1.7e-7), which consisted of 36 phenotypes (phenotype number). **Supplementary Table 5.** meQTLs trait enrichment using associates downloaded from GWAS Catalog (https://www.ebi.ac.uk/gwas/). MeQTLs for cocaine-related DNA methylations were used. **Supplementary Table 6.** Candidate CpG sites for cocaine use frequency (high frequency users VS low frequency users). **Supplementary Table 7.** MeQTLs (FDR < 0.05) for cocaine use frequency-related DNA methylations after meta-analysis to combine results for the 450K and EPIC cohorts (Ntotal = 293) and clumping. **Supplementary Table 8.**  Enriched Ingenuity Canonical Pathways identified using genes mapped by the meQTLs for cocaine use frequency-related DNA methylations. **Supplementary Table 9.** Significant traits associated with the top index meQTLs for cocaine use frequency-related DNA methylations. A total of 4 studies reached the significance level after bonferroni correction (p < 7.0e-7), which consisted of 4 phenotypes (phenotype number). **Supplementary Table 10.** meQTLs trait enrichment using associates downloaded from GWAS Catalog (https://www.ebi.ac.uk/gwas/). MeQTLs for cocaine use frequency-related DNA methylations were used.

## Data Availability

Demographic and clinical variables and DNAm data for the VACS samples were submitted to GEO dataset (GSE117861) and are publicly available. All codes for analysis are also available upon a request to the corresponding author.
