# Supplementary material for: Cis-meQTL for cocaine use-associated DNA methylation in an HIV-positive cohort show pleiotropic effects on multiple traits
Source: BMC Genomics. 2023 Sep 20;24:556. doi: 10.1186/s12864-023-09661-2 (PMC10510240; doi:10.1186/s12864-023-09661-2)
Supplement: Supplementary file 1 — Additional file 1: Supplementary Figure 1. Global ancestry estimates by ADMIXTURE. 2,504 residents with African (AFR), East Asian (EAS), European (EUR), and South Asian (SAS) ancestry from the 1000 Genomes Project were used as the reference genotype panel to infer the super populations membership. Results were plotted for (A) samples in the Veterans Aging Cohort Study (VACS) cohort (n = 2244) with respect to reference samples, (B) a subset of the VACS cohort with admixed ancestral information (n = 105) with respect to reference samples. The reference and VACS samples were separated by the black line. Supplementary Figure 2. Inferred global ancestry of the VACS samples. Scatter plot of the genotype principal component analysis (PCA) results (PC1 and PC2) for the VACS cohort and 1000 Genome Project were plotted. The color indicated the super population of 1000 Genome reference samples (dots), and the inferred global ancestry of the VACS samples (triangles). Supplementary Figure 3. Selection of candidate CpGs associated with cocaine use in the Veterans Aging Cohort Study (VACS) samples. (A) Manhattan plot and (B) QQ plot (genomic inflation λ = 1.196) after meta-analysis to combine results from the 450K and EPIC cohorts. A total of 224 candidate CpG sites were identified. The red line indicates the p-value threshold used to identify candidate CU-associated CpG sites (p-value < 0.0001). Supplementary Figure 4. Two representative patterns of genetic effects by cocaine use for the meQTLs identified. (A-B): the distribution of methylation by the genotype among cocaine non-users and users. The patterns in the 450K cohort and EPIC cohort were plotted separately. (A) The genetic effect of rs13233191 on the methylation of cg17914838. (B) The genetic effect of rs7834638 on the methylation of cg21175976. CU: cocaine use; meQTL: methylation quantitative trait loci. [file 12864_2023_9661_MOESM1_ESM.doc]

**
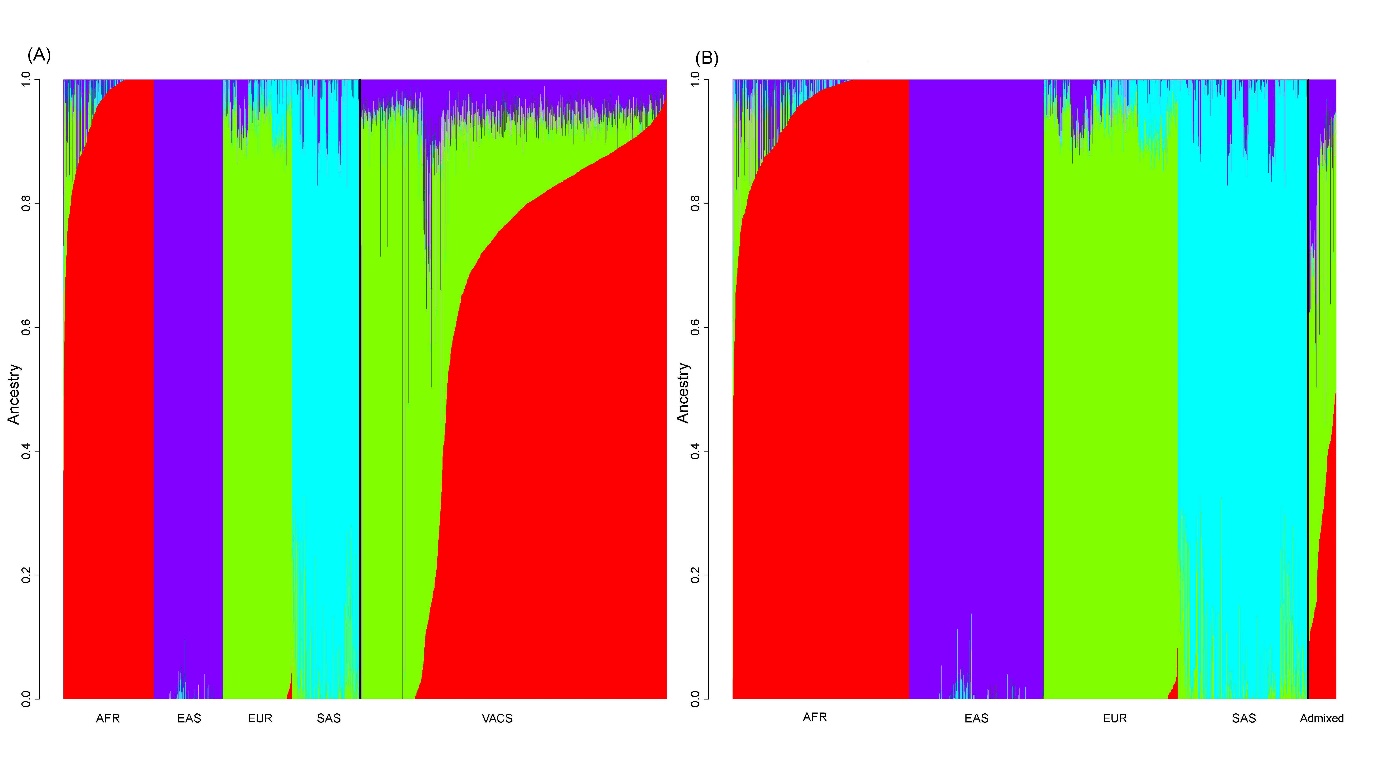
**

**Supplementary Figure 1.** **Global ancestry estimates by ADMIXTURE.** 2,504 residents with African (AFR), East Asian (EAS), European (EUR), and South Asian (SAS) ancestry from the 1000 Genomes Project were used as the reference genotype panel to infer the super populations membership. Results were plotted for (A) samples in the Veterans Aging Cohort Study (VACS) cohort (n = 2244) with respect to reference samples, (B) a subset of the VACS cohort with admixed ancestral information (n = 105) with respect to reference samples. The reference and VACS samples were separated by the black line.

**
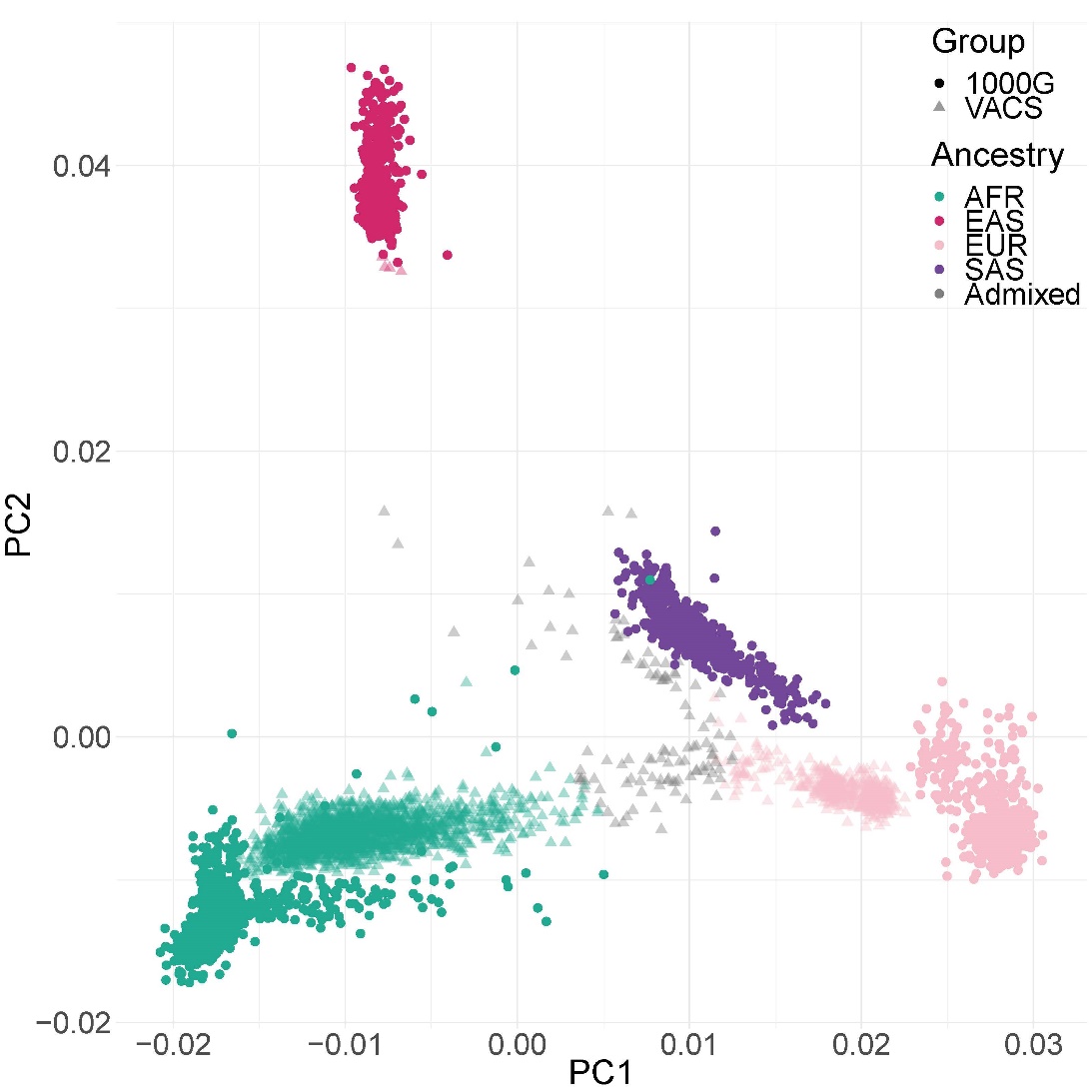
**

**Supplementary Figure 2.** **Inferred global ancestry of the VACS samples.** Scatter plot of the genotype principal component analysis (PCA) results (PC1 and PC2) for the VACS cohort and 1000 Genome Project were plotted. The color indicated the super population of 1000 Genome reference samples (dots), and the inferred global ancestry of the VACS samples (triangles).


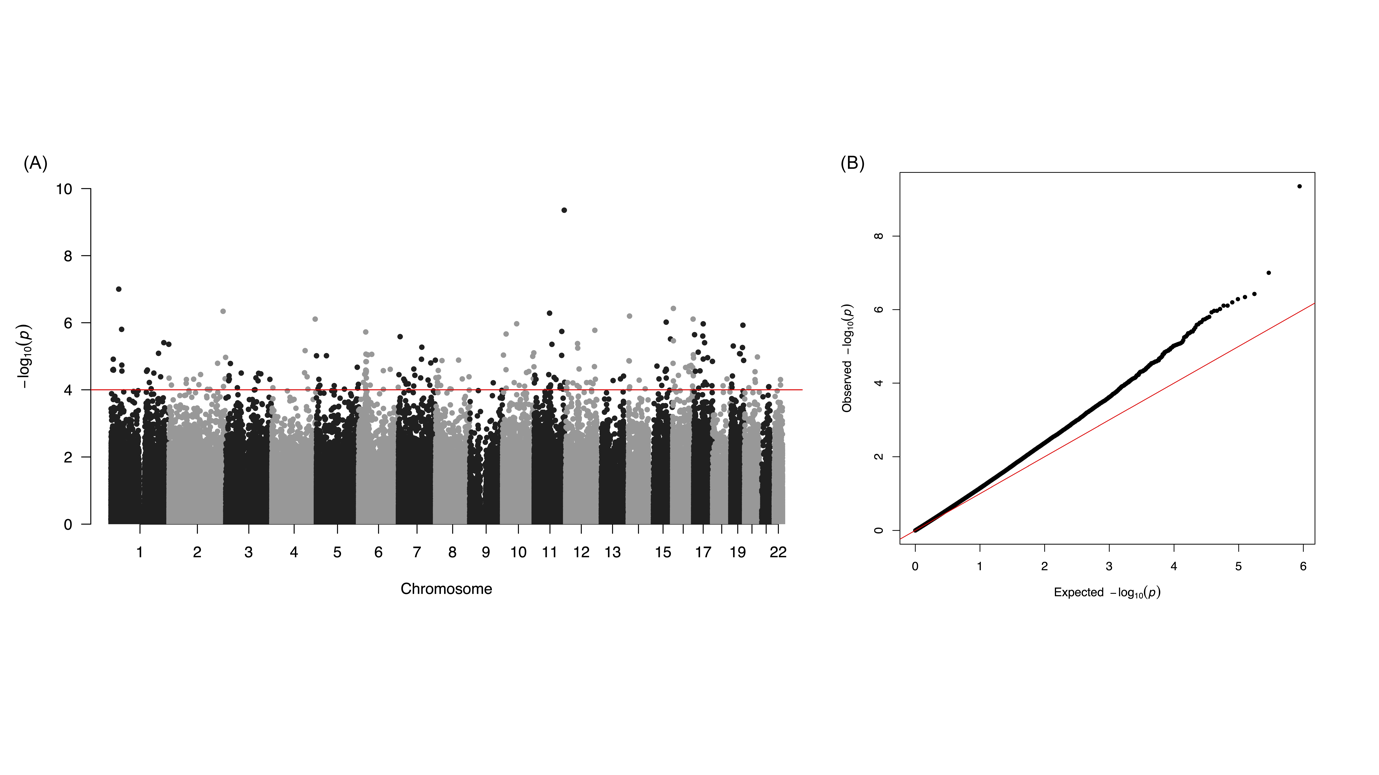


**Supplementary Figure 3. Selection of candidate CpGs associated with cocaine use in the Veterans Aging Cohort Study (VACS) samples.** (A) Manhattan plot and (B) QQ plot (genomic inflation λ = 1.196) after meta-analysis to combine results from the 450K and EPIC cohorts. A total of 224 candidate CpG sites were identified. The red line indicates the p-value threshold used to identify candidate CU-associated CpG sites (p-value < 0.0001).

**
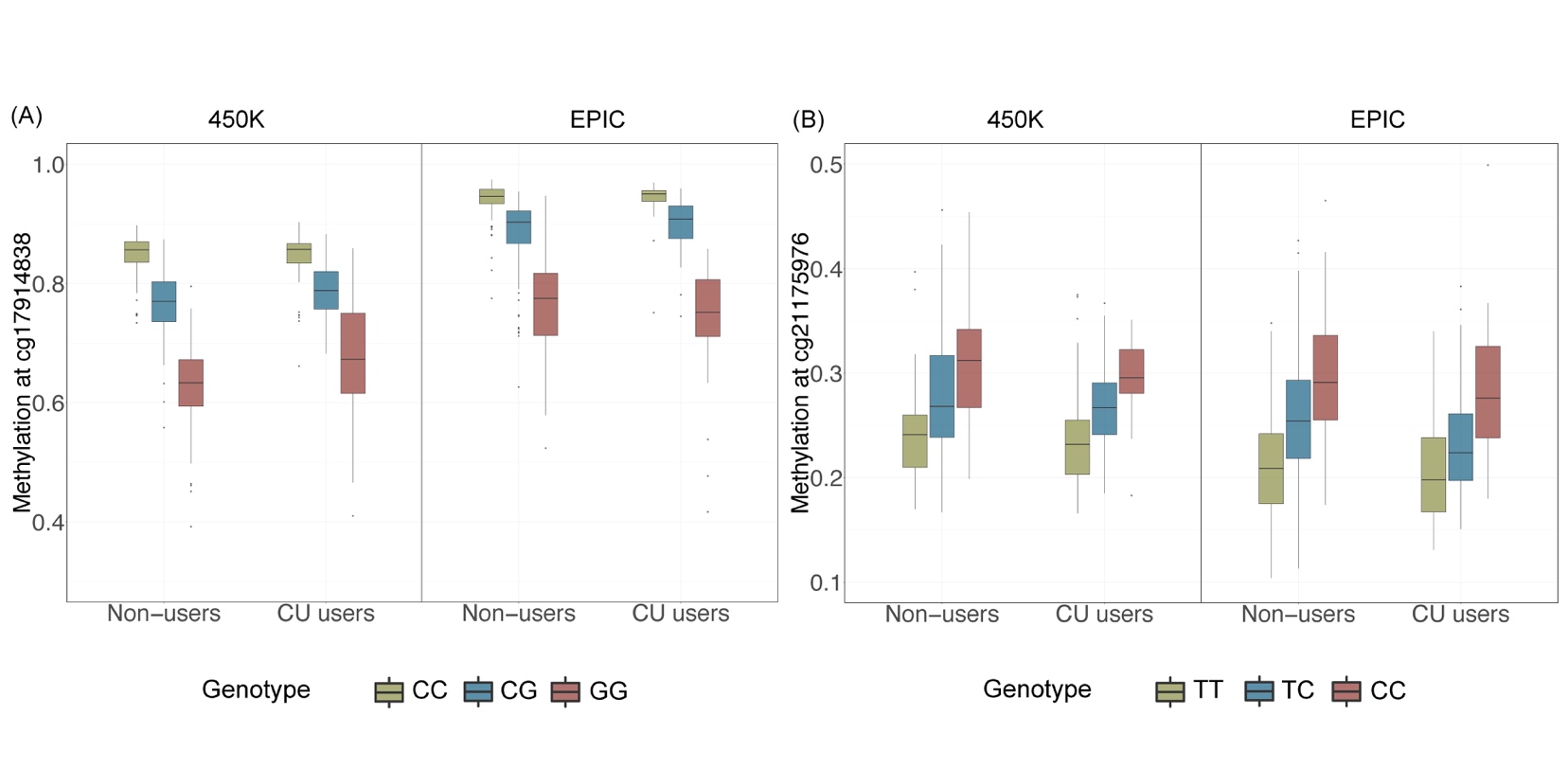
**

**Supplementary Figure 4.** **Two representative patterns of genetic effects by cocaine use for the meQTLs identified.**

(A-B): the distribution of methylation by the genotype among cocaine non-users and users. The patterns in the 450K cohort and EPIC cohort were plotted separately.

(A) The genetic effect of rs13233191 on the methylation of cg17914838. (B) The genetic effect of rs7834638 on the methylation of cg21175976.

CU: cocaine use; meQTL: methylation quantitative trait loci.
